# A Novel Phosphorylated Tau Conformer Implicated in the Tauopathy Pathogenesis of Human Neurons

**DOI:** 10.3390/biom15040585

**Published:** 2025-04-15

**Authors:** Nahid Tofigh, Sadaf Agahi, Gholamhossein Riazi, Mahboobeh Ghalamkar Moazzam, Koorosh Shahpasand

**Affiliations:** 1 Laboratory of Neuro-Organic Chemistry, Institute of Biochemistry and Biophysics (IBB), University of Tehran, Tehran 13561-457, Iran; tofigh@ut.ac.ir; 2 Department of Brain and Cognitive Sciences, Cell Science Research Center, Royan Institute for Stem Cell Biology and Technology, ACECR, Tehran 16635-148, Iran; moazzamgh@gmail.com; 3 Department of Medicine, School of Medicine, Tehran University of Medical Sciences, Tehran 14768-211, Iran; agahi.sadaf25@gmail.com; 4 Department of Laboratory Medicine and Pathology, Medical School, University of Minnesota, Minneapolis, MN 55414, USA

**Keywords:** Alzheimer’s disease, pT231-tau monoclonal antibody, neurodegeneration, gauche p-tau

## Abstract

Alzheimer’s disease (AD) is a neurodegenerative disorder with no effective treatments. Hyperphosphorylation of tau protein contributes to neurodegeneration in AD. Previous studies have identified pT231-tau in the cis conformation as an early driver of neurodegeneration in tauopathy models. Here, we identify a novel neurotoxic pT231-tau conformer in human AD neurons, distinct from both cis and trans conformations, which we propose as the gauche pT231-tau conformer. Notably, levels of this conformer were elevated in neurons subjected to aging-associated stress. In order to confirm the stress, we examined p21 accumulation in both human iPSC-derived and mouse cortical neurons under aging stress. Targeted elimination of the gauche pT231-tau conformer mitigated neurodegeneration in human AD cultures. These findings suggest the gauche pT231-tau conformer plays a key role in tau-mediated neurodegeneration and may be a potential therapeutic target for AD.

## 1. Introduction

Alzheimer’s disease (AD) is a chronic neurodegenerative disorder and the sixth leading cause of death in the United States [1]. It is characterized by two hallmark neuropathological features: neurofibrillary tangles (NFTs), composed of hyperphosphorylated tau protein, and senile plaques, formed by β-amyloid peptide [2,3,4]. Tau hyperphosphorylation leads to its aggregation into NFTs, a key feature of AD and other tau-related neurodegenerative disorders collectively referred to as tauopathies [5,6,7,8]. Pathogenic tau exhibits prion-like properties, spreading across brain regions and exacerbating disease progression [9,10,11,12,13,14,15]. Prion-like properties refer to the ability of tau proteins to misfold and induce pathological conformations in other tau molecules, thereby propagating neurotoxicity in a templated manner.

Among the many phosphorylation sites on tau, threonine 231 (Thr231) phosphorylation plays a pivotal role in both tau function and pathology. Recent studies have identified two distinct conformations of phosphorylated tau at Thr231: cis and trans. The cis conformer acts as an early driver of neurodegeneration, promoting microtubule destabilization and neuronal toxicity, while the trans conformer is considered a physiological state without significant pathological accumulation [16,17,18,19,20,21]. The enzyme peptidyl prolyl isomerase 1 (Pin1) mediates the interconversion between these conformers, facilitating the transition from cis to trans pT231-tau [22,23].

Our previous studies demonstrated that traumatic brain injury (TBI) leads to a pathological increase in cis pT231-tau, disrupting microtubule stability and mitochondrial transport, ultimately resulting in apoptosis—a phenomenon termed cistauosis [17,24]. Notably, eliminating cis pT231-tau suppresses tau pathology and cognitive decline in TBI mouse models [25,26,27]. Conversely, trans pT231-tau does not accumulate in TBI mouse brains and is considered a physiologic conformer [28,29]. These findings have established cis pT231-tau as a neurotoxic factor in both mouse and human neurons [17,30].

This study extends beyond previous work on cis pT231-tau by identifying gauche pT231-tau as a distinct pathogenic conformer associated with aging-related tauopathy in AD, rather than traumatic brain injury. This highlights a previously unrecognized mechanism of tau pathology, offering new insights into the role of tau conformers in neurodegeneration and opening avenues for targeted therapeutic strategies.

Despite these insights, tau pathology may exhibit species-specific differences between mice and humans, raising questions about whether additional pathogenic tau conformers exist in the human brain. To address this, we examined various conformation-specific pT231-tau antibodies in both human and mouse neurons under aging-related stress conditions. While cis pT231-tau is a known pathogenic driver of tauopathy, our study identifies a previously unrecognized conformer, which we termed gauche pT231-tau, that accumulates specifically in human AD neurons. Unlike cis pT231-tau, which is primarily linked to traumatic brain injury, gauche pT231-tau is uniquely associated with aging-related tau pathology in AD. Structural constraints may have prevented its detection in earlier cis–trans-focused studies. These findings suggest that gauche pT231-tau represents a distinct neurotoxic entity, highlighting the need for targeted therapeutic intervention.

## 2. Materials and Methods

### 2.1. Human NPC Generation and Differentiation

Human-induced pluripotent stem cells (hiPSCs) were obtained from the Royan cell bank. hiPSC clones were separated from mouse embryonic fibroblasts using a cocktail of 50 mg/mL collagenase (Gibco, USA) and 100 mg/mL dispase (Thermo Fisher Scientific, USA) through a 5 min incubation at 37 °C with 5% CO_2_. Cells were cultured in a human embryonic stem cell medium consisting of Dulbecco’s modified Eagle’s medium (DMEM/F12; Gibco, 12634010), 20% knockout serum (KOSR; Gibco, 10828-028), 1% non-essential amino acids (NEAAs; Gibco, 11140068), 1% L-glutamine (2 mM; Invitrogen, USA, 25030-024), 1% penicillin/streptomycin (Gibco, 15140148), 1% insulin transferrin selenium (ITS; Gibco, 1898732), and basic fibroblast growth factor (bFGF; 100 ng/mL final concentration; Royan Institute, Tehran, Iran). The cells were cultured on mitotically inactivated mouse embryonic fibroblasts (MEFs), which provide essential support for their growth and maintenance. The culture dishes were coated with Matrigel (Sigma-Aldrich, USA, E1270) and kept in an incubator at 37 °C with 5% CO_2_, with the medium refreshed daily. After 7 days, the medium was replaced with an induction medium supplemented with 1% N2 supplement (Gibco, LS17502048), 0.1% B27 supplement (Gibco, 12587010), and the following small molecules at final concentrations: 3 μM CHIR99021 (Stemgent, USA, S04000410), 3 μM SB4315242 (Cayman, USA, 13031), 5 μM dorsomorphin (Stemgent, 040024), and 0.5 μM SAG (Calbiochem, USA, 566660). Medium changes were performed every other day for 7 days. Rosettes formed during this period were mechanically dissociated into single cells using trypsin/EDTA (Gibco, 25300054) and transferred to culture plates coated with laminin (1 mg/mL; Sigma-Aldrich, L2020) and poly-L-ornithine (15 mg/mL; Sigma-Aldrich, P4957). Cells were then cultured in DMEM/F12 medium supplemented with 5% KOSR, 1% NEAAs, 1% L-glutamine, 1% penicillin/streptomycin, 1% N2, 1% ITS, and bFGF (40 ng/mL final concentration; Royan Institute, Tehran, Iran), referred to as the neural expansion medium. Medium was refreshed every 2 days, and cells were passaged upon reaching high confluency. Neural differentiation was performed using a medium containing DMEM/F12, 5% KOSR, 1% N2 supplement, 1% B27 supplement, 1% L-glutamine, 1% penicillin/streptomycin, and 1% NEAAs, along with the following factors: brain-derived neurotrophic factor (BDNF; 20 ng/mL; PeproTech, Netherlands), glial cell line-derived neurotrophic factor (GDNF; 20 ng/mL; PeproTech), ascorbic acid (0.2 mM; Sigma), and dibutyryl cAMP (5 μg/mL; Sigma). Half of the medium was refreshed every 3 days [31,32]. Treatments were administered on day 27.

### 2.2. Mouse Primary Cortical Neuron Culture

Primary cortical neuron cultures were established using cerebral cortex tissues from C57BL/6 mouse embryos at embryonic days 15–16 (E15–16), following previously established protocols [17]. All animal experiments were conducted in accordance with the ethical recommendations outlined in the Guide for the Care and Use of Laboratory Animals (National Institutes of Health Publication No. 80-23, revised 1996) and were approved by the Institutional Research Ethics Committee (IR.ACECR.ROYAN.REC.1402.087). Pregnant mice were sacrificed via cervical dislocation, and the uterus was dissected to collect six to eight embryos. Cortical tissues were isolated and subjected to enzymatic digestion using trypsin/EDTA (Gibco, 25300054), DNase I (Roche, USA, 11284932001), HEPES (Sigma, USA, H0887), and D-glucose (Sigma, USA, D6134). Culture plates and dishes were pre-coated with a combination of poly-L-ornithine and laminin before cell plating. Cells were seeded at densities of 1.5 × 10^4^ and 6 × 10^5^ cells/mL, respectively, and cultured in a neurobasal medium (Gibco, 21103049) supplemented with 2% B-27, 1% L-glutamax, and 1% penicillin/streptomycin. Cultures were maintained at 37 °C in a 5% CO_2_ incubator, with half of the culture medium refreshed every 2 days. Treatments were initiated on day 7 in culture. Aging-related stress in mouse cortical neurons was induced by prolonged culturing (21 days in vitro), consistent with established in vitro protocols [17].

### 2.3. Live and Dead Cell Assay

To evaluate neuronal viability, human-induced pluripotent stem cell-derived neural progenitors (hiPSC-NPs) were seeded into 4-well culture plates at a density of 1.5 × 10^5^ cells per well and allowed to undergo differentiation for 27 days. Similarly, mouse primary cortical neurons were seeded on the same plates and differentiated for 7 days. The stress induction protocols in hiPSC-derived neural progenitors (hiPSC-NPs) and mouse primary cortical neurons were somehow distinct, with age-dependent stress factors being considered for each model. For the hiPSC-NP model, we assessed 45-day and 27-day-old cultures to evaluate the effects of stress on p21 accumulation. These time points were selected based on previous studies showing p21 accumulation as a marker of neuronal senescence. Similarly, stress was induced in mouse primary cortical neurons through exposure to aging-related stress (21-day and 7-day), allowing for comparison between the two models. The rationale for these protocols and the choice of time points is discussed in the context of the existing literature on aging-related stress induction in neuronal models. Cells were maintained at ~80% confluence with media changes every 48 h. Media color and pH were monitored to prevent nutrient depletion and pH drift.

Following differentiation, neuronal stress was induced by aging, and cells were treated with monoclonal antibodies against cis, trans, and gauche pT231-tau (Table 1). Cis and trans pT231-tau antibodies were included to serve as benchmarks for comparison with the newly identified gauche pT231-tau conformer, allowing for the assessment of its distinctiveness from known pathological tau forms. These antibodies were administered at the onset of stress, with final concentrations of 0.009 and 0.0045 µM for anti-cis pT231-tau and 0.06 and 0.03 µM for anti-gauche pT231-tau, over a treatment period of 96 h. The goal was to assess the viability of the treated neurons. Neuronal viability was assessed using live/dead staining, employing fluorescein diacetate (FDA) to label viable cells and propidium iodide (PI) to label dead cells. A working solution was prepared by diluting 20 µL of FDA stock and 50 µL of PI stock in 10 mL of PBS, following the method described by Jiajia et al. [33]. Prior to staining, the cells were rinsed with cold PBS. The FDA/PI solution was then applied to the cells and incubated at room temperature for 5 min. The stained cells were visualized using a fluorescence microscope (IX71, Olympus, Tokyo, Japan) for analysis.

### 2.4. Visualization of Microtubule Structure

Human neural progenitor cells (NPCs) were differentiated in the absence of bFGF and cultured for 27 days. After this period, the cells were subjected to neuronal aging stress. As part of this stress, the cells were treated with monoclonal antibodies (mAbs) against gauche pT231-tau (0.06 µM) and cis pT231-tau (0.009 µM) for 96 h. Following treatment, the cells were stained to visualize the microtubule structure using a tubulin antibody and observed under a confocal microscope. The staining procedure involved washing the cells and incubating them with 0.5% Triton X-100 in PBS for 10 min to permeabilize the cytoplasm. The cells were then fixed with pre-chilled methanol (−20 °C) for 15 min. After fixation, the cells were rinsed with PBS and incubated with a tubulin antibody (BioLegend, USA, 801213, 1 μg/mL) for 1 h at room temperature. A secondary antibody (anti-mouse Alexa Fluor 568, Invitrogen, USA, A-11057, 2 μg/mL) was applied for 1 h at 37 °C. Nuclei were stained using DAPI. Once the immunofluorescent staining was complete, coverslips were mounted onto glass slides, and microtubule visualization was conducted using a Zeiss confocal microscope, LSM 800, SCR_015963.

### 2.5. Immunoblotting

Either postmortem human brain samples or cultured cells were lysed using RIPA buffer (Sigma-Aldrich, USA, MFCD02100484), supplemented with phosphatase inhibitors (Sigma-Aldrich, USA, P5726) and a cocktail of protease inhibitors (Sigma-Aldrich, USA, P2714). After centrifugation at 10,000× *g* for 20 min at 4 °C, the supernatant was collected. The protein concentration was determined using the bicinchoninic acid (BCA) method (Thermo Fisher Scientific, Rockford, IL, USA). Protein samples were mixed with SDS-containing buffer and heated to 95 °C for 5 min. Then, 20 μg of protein was loaded onto a 12% SDS-PAGE gel. Proteins were separated by SDS-PAGE, then transferred to a polyvinylidene fluoride (PVDF) membrane (Biorad, USA, 1620177). The membrane was blocked with 2% bovine serum albumin (BSA; Roche, USA, 9048-46-8) in TBST (10 mM Tris-HCl pH 7.6, 150 mM NaCl, 0.1% Tween-20) for 1 h at room temperature. After blocking, the membrane was incubated overnight at 4 °C with the following primary antibodies: cis pT231-tau monoclonal antibody (gift from Prof. KP Lu, Harvard, RRID: AB_2877630, mouse monoclonal, 1:2500), gauche pT231-tau monoclonal antibody (1:1500), Tau5 (Millipore, USA, mouse monoclonal, 1:1000), and anti-β-actin (loading control; Proteintech, USA, IG-60008-1, 1: 30,000). The membrane was washed three times for 15 min each with 0.1% Tween-20 TBS (pH 7.6), then incubated with secondary antibodies: anti-mouse or anti-mouse monoclonal c-Myc horseradish peroxidase (HRP)-conjugated secondary antibody (Santa Cruz, CA, USA, H2317) for 1 h at room temperature.

After three additional 10 min washes, protein bands were visualized using an enhanced chemiluminescence (ECL) reagent (Amersham Biosciences, RPN2232, USA). β-actin served as the housekeeping protein for normalization. Quantitative densitometry analysis of the protein bands was performed using ImageJ (version 1.54p software).

### 2.6. Immunodepletion Experiment

Gauche monoclonal antibodies (Gauche mAbs) were immobilized on cyanogen bromide-activated Sepharose to deplete gauche pT231-tau from the sample extract. Sepharose powder (1 g) was suspended in 1 mM HCl and incubated for 30 min at room temperature, followed by a 15 min wash with cold 1 mM HCl. The gauche mAb was dissolved in a coupling buffer (0.1 M NaHCO_3_, pH 8.3, 0.5 M NaCl) and added to the Sepharose suspension. The mixture was gently rotated and incubated overnight at 4 °C to allow chemical coupling of the mAb to the resin. After incubation, excess mAb was washed away twice with 5 mL of coupling buffer. To block the remaining active groups on the Sepharose, the resin was incubated with 0.1 M Tris/HCl buffer (pH 8.0) for 2 h. The resin was subsequently washed through three cycles of alternating Tris/NaCl and glycine/NaCl buffers. Finally, the extract mixed with PBS was added to the resin column to deplete the gauche pT231-tau. Immunoblotting analysis was performed as described above.

### 2.7. Cell Immunostaining

Neurons were fixed with 4% (*w*/*v*) paraformaldehyde for 20 min at room temperature. Following fixation, the cells were washed twice with PBS-Tween at 5 min intervals. Next, cells were permeabilized with 0.5% Triton X-100 for 10 min, then blocked with 1% (*w*/*v*) BSA for 1 h. After a final wash with PBS-Tween, the cells were incubated with primary antibodies (cis, trans, and gauche P-tau mAbs) overnight at 4 °C. The following day, the cells were washed three times with PBS-Tween and incubated with c-Myc antibody (for gauche P-tau mAb) for 2 h. Afterward, the cells were treated with goat anti-mouse secondary antibody (Alexa Fluor 594; Invitrogen, USA, A-11020) at 37 °C for at least 1 h. To assess the penetrance of the gauche p-tau mAb, cell plates were treated or untreated with the mAbs overnight. The cells were then fixed and stained with c-Myc primary antibody followed by the respective secondary antibody for 1 h. The cell nuclei were counterstained with 4,6-diamidino-2-phenylindole (DAPI; Sigma, USA, D8417) for 2 min at room temperature. Cells were visualized using an Olympus IX71 microscope equipped with an Olympus DP72 digital camera. Image analysis was performed using ImageJ software.

### 2.8. Immunofluorescence Staining of Human Brain Sections

Immunofluorescence techniques were employed to examine postmortem brain sections obtained from three patients in the late stages of Alzheimer’s disease (AD) and three age-matched healthy controls. The human brain tissues were provided by Iranian forensic medicine organizations.

Initially, the tissues were deparaffinized in two rounds using xylene, with each round lasting 10 min. Following deparaffinization, the sections were rehydrated by sequential immersion in ethanol dilutions (100%, 90%, and 70%) for 3 min each, followed by immersion in deionized water. To minimize autofluorescence, the sections were quenched overnight with 5% ammonium chloride.

Antigen retrieval was performed by boiling the sections in a 10 mM sodium citrate solution (pH 6.0) for 20 min. Afterward, the sections were treated with 10% hydrogen peroxide for 30 min. After thorough washing with 0.05% PBS-T, the sections were permeabilized with 0.5% Triton-X for 10 min, blocked with 1% *w*/*v* BSA for 1 h, and then incubated overnight at 4 °C with cis, trans, and gauche P-tau monoclonal antibodies as primary antibodies.

In the case of gauche mAb staining, after three 15 min washes with 0.05% PBS-T, the sections were incubated with c-Myc antibody for 2 h, followed by incubation with the respective secondary antibody conjugated to Alexa Fluor 594 for 1 h at room temperature in the dark. The slides were then washed twice with 0.05% PBS-T for 5 min each. The cell nuclei were counterstained with 4,6-diamidino-2-phenylindole (DAPI) (Invitrogen, D1306). The stained sections were visualized using a fluorescent microscope (Olympus, BX51, equipped with an Olympus DP72 digital camera). Finally, the acquired images were analyzed using ImageJ 1.54p software.

### 2.9. Tertiary Structural Prediction of Human and Mouse Tau Protein

The I-TASSER (Iterative Threading Assembly Refinement) server, an online tool specialized in automatic protein tertiary structure prediction through the threading method, was utilized [35,36,37]. To begin the process, the primary amino acid sequences of full-length tau proteins were provided in FASTA format: 441 residues for human tau and 430 residues for mouse tau. The I-TASSER server then performed modeling simulations, generating five structural models along with their corresponding PDB files, all of which exhibited relatively high confidence levels. For further analysis, we selected the initial model with the higher *p*-score, which we deemed to have superior quality. The PDB-formatted structure files for both human tau (*p*-score = 0.35) and mouse tau (*p*-score = 0.38) were obtained. The amino acids within these structures were visualized using PyMOL (version 1.20) (Figure 7a,b).

### 2.10. ELISA Analysis

To evaluate the binding affinities and specificities of our antibodies, we performed enzyme-linked immunosorbent assay (ELISA)-based titrations using synthetic peptides representing the cis, trans, and gauche pT231-tau conformers. The synthetic peptides used were as follows:

Cis pT231-tau: V-A-V-V-R-pT-(Pip)-P-K-S-P;

Trans pT231-tau: V-A-V-V-R-pT-A-P-K-S-P;

Gauche pT231-tau: V-A-V-V-R-pT-G-P-K-S-P.

96-well high-binding polystyrene plates (Nunc MaxiSorp) were coated with 100 μL of each synthetic peptide (5 μg/mL) in carbonate-bicarbonate buffer (pH 9.6) and incubated overnight at 4 °C. Wells were blocked with 5% bovine serum albumin (BSA) in PBS-T (PBS with 0.05% Tween-20) for 1 h at room temperature to prevent nonspecific binding. Serial dilutions of the anti-pT231-tau antibodies were prepared in blocking buffer and added to the wells (100 μL/well), followed by incubation for 2 h at room temperature with gentle shaking. After washing three times with PBS-T, HRP-conjugated secondary antibody was added at a 1:5000 dilution and incubated for 1 h at room temperature. Wells were washed again, and TMB substrate solution (3,3′,5,5′-tetramethylbenzidine) was added for colorimetric detection. The reaction was stopped with 2N sulfuric acid, and absorbance was measured at 450 nm using a microplate reader (BioTek Synergy H1,USA). Absorbance values were plotted against antibody concentrations to generate binding curves. Affinity differences were quantified by calculating the half-maximal effective concentration (EC_50_).

### 2.11. Statistical Analysis

For the data presented in Figures 4d, 5b and Supplementary Figures S3c and S4c, a one-way ANOVA was performed, followed by a post hoc Tukey’s test. For all other statistical comparisons, a two-tailed unpaired Student’s *t*-test was used to compare specific pairs of groups, as indicated in the figure legends. Statistical significance was defined as a *p*-value < 0.05. Data are presented as mean values ± SEM with a 95% confidence interval. Data analysis and visualization were conducted using GraphPad Prism version 7. To assess the co-localization of cis/gauche or trans/gauche p-tau, we conducted quantitative co-localization analysis using Pearson’s correlation coefficient.

## 3. Results

To investigate the role of gauche pT231-tau in tauopathy, we employed a multi-model approach using human postmortem AD brains, iPSC-derived neurons, and primary mouse cortical neurons. Postmortem brains provide direct pathological validation, iPSCs allow controlled in vitro modeling, and mouse neurons offer mechanistic insights. This integrative approach strengthens our understanding of gauche pT231-tau’s role in neurodegeneration.

### 3.1. Neurons Were Generated from AD and Healthy *iPSC*s

To assess the relative toxicity of cis and gauche P-tau conformers in human Alzheimer’s disease (AD) neurons, human-induced pluripotent stem cells (hiPSCs) were derived from five patients with early-onset Alzheimer’s disease (AD3) and five age-matched healthy control subjects, obtained from the Royan Cell Bank (Supplementary Figure S1b). Early-onset AD patient-derived iPSCs were selected because they exhibit more aggressive tau pathology, which offers a more sensitive platform for detecting pathogenic tau conformers. This choice allows for a more robust examination of tau conformer dynamics in a model that closely reflects the accelerated disease progression seen in early-onset AD. Human iPS4 cells were generated from healthy age-matched control subjects. These iPSCs were derived from non-diseased individuals of similar age to the AD patient-derived iPSCs, serving as the control group for comparison in the study. The suspended iPSC colonies were cultured on Matrigel-coated dishes, and iPSCs were subsequently transitioned into a neural induction medium to promote rosette formation. These rosettes were then transferred onto dishes coated with laminin and poly-L-ornithine, where they were cultured in a neural progenitor cell (NPC) medium. Over time, they differentiated into a mixed population of neurons (Supplementary Figure S1a,b). The cells derived from hiPSCs stained positive for SOX2, PAX6, and Nestin, confirming their identity as NPCs. Neuronal differentiation was verified by immunostaining with neuronal markers, including GABA, GAD, and MAP2 (Supplementary Figure S2).

### 3.2. A Significant Accumulation of p21 Was Observed in Neurons as a Result of Aging

Several studies have reported a direct link between aging and the accumulation of p21 (inhibitor of cyclin-dependent kinases, CDKN1A) [38]. Mature postmitotic neurons accumulate various types of DNA damage with age. This damage triggers the DNA damage response (DDR), which is the primary initiator of senescence mechanisms. p21, a cyclin-dependent kinase inhibitor, acts as a signal transducer between the DDR and senescence-associated characteristics in neurons. Cells exhibiting a senescent phenotype are often marked by elevated levels of p21. Moreover, a correlation between aging and tauopathy has been demonstrated in several studies [39,40,41,42,43]. p21 is a key marker of neuronal senescence, which is implicated in aging-related neurodegeneration, including tauopathy. Its potential role in driving tau pathology prompted us to assess p21 accumulation in human and mouse neurons under aging stress, providing a foundation for subsequent experiments on tau conformer accumulation in aged neurons. In our study, we observed an increase in cell senescence in both mouse primary cortical neurons and hiPSC-derived neurons under aging stress conditions, as indicated by the accumulation of p21. Specifically, during the first 27 days in vitro (27D), human neurons reach maturity [44]. while mouse primary cortical neurons mature over the first seven days of culture (7D). As the cultures age, neuronal senescence is observed [45]. As shown in Supplementary Figure S3a,c, neuronal aging in human neurons (45D) resulted in significantly higher p21 levels compared to control mature neurons (27D) (21 ± 2.08 vs. 1.33 ± 0.33, *n* = 3, *p* = 0.0221), indicating cellular senescence. We further confirmed this finding by measuring p21 levels through immunoblotting, which revealed a significant increase in p21 in aged neurons (1.38 ± 0.07 vs. 0.8641 ± 0.01, *n* = 3, *p* = 0.002) (Supplementary Figure S3b,d).

Similarly, aged mouse neurons (21D) exhibited significantly higher p21 levels compared to control mature neurons (7D) (12.33 ± 1.45 vs. 0.67 ± 0.33, *n* = 3, *p* = 0.0004), confirming neuronal senescence (Supplementary Figure S4a,c). Immunoblotting analysis also showed a marked increase in p21 levels in aged mouse neurons under age-related stress (1.379 ± 0.21 vs. 0.419 ± 0.09, *n* = 3, *p* = 0.015) (Supplementary Figure S4b,d).

### 3.3. Postmortem Brains from Alzheimer’s Disease (AD) Patients Exhibited a Significant Increase in the Gauche pT231-Tau Conformer Compared to Healthy Control Subjects

**Figure 1:**
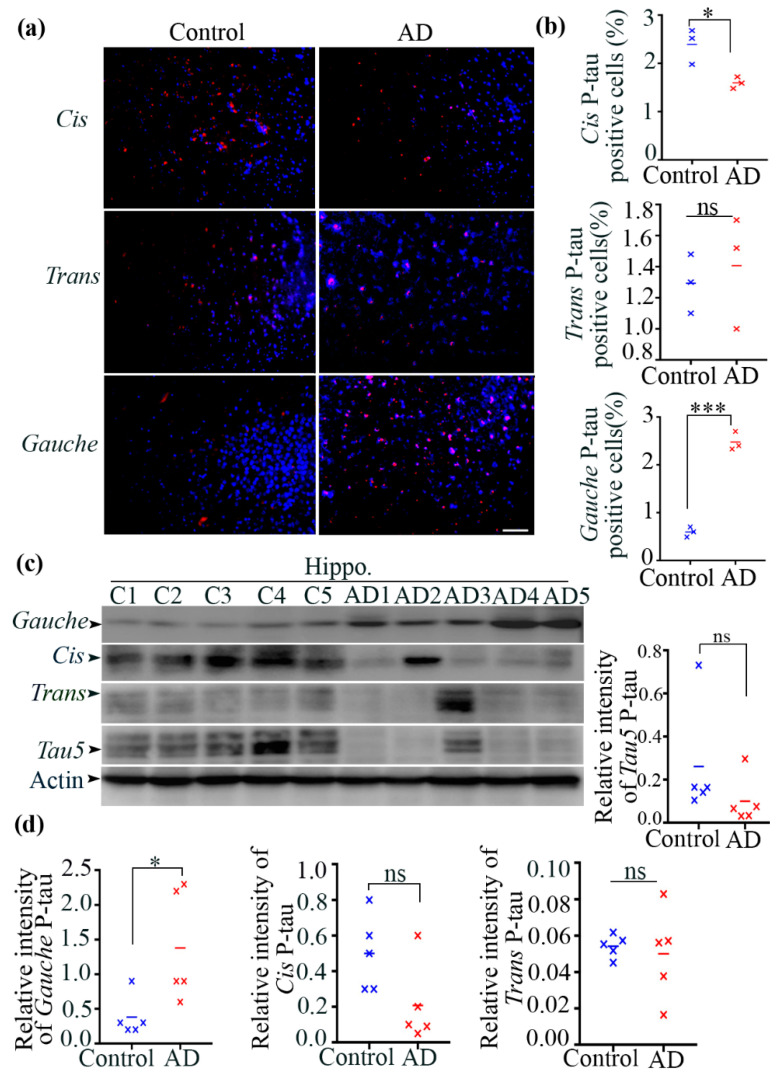
Accumulation of gauche P-tau in human AD brains. (**a**) Immunofluorescent images of the human hippocampus from AD and control brains. (**b**) Quantification of cis, trans, and gauche P-tau levels. Data are presented as mean ± SEM; *n* = 3 participants; * *p* < 0.05 and *** *p* < 0.001 by unpaired t-test. Scale bars: 200 μm. (**c**) Immunoblots of tau5, cis, trans, and gauche P-tau levels in hippocampal lysates from healthy (C1–C5) and AD brains (AD1–AD5). (**d**) Quantification of tau5, cis, trans, and gauche P-tau levels. Data are presented as mean ± SEM; *n* = 5 participants; n.s.: not significant; * *p* < 0.05 by unpaired *t*-test. Original Western blot images can be found in Figure S8.

Previous studies have reported an increase in cis P-tau in traumatic brain injury (TBI) and chronic traumatic encephalopathy (CTE) [15,17,46,47]. In this study, we examined the accumulation of cis, trans, and gauche P-tau in the hippocampal regions of postmortem brains from Alzheimer’s disease (AD) patients and age-matched healthy controls (Table 2). Immunostaining with anti-cis, trans, and gauche P-tau antibodies revealed alterations in cis P-tau (1.597 ± 0.06 vs. 2.393 ± 0.21, *n* = 3, *p* = 0.0233) and trans P-tau (1.407 ± 0.209 vs. 1.293 ± 0.109, *n* = 3, *p* = 0.6572) levels in AD brains compared to controls (Figure 1a). Importantly, there was a significant increase in gauche P-tau in AD brains compared to controls (2.477 ± 0.11 vs. 0.5967 ± 0.06, *n* = 3, *p* = 0.0001) (Figure 1b). In our analysis, we considered a result to be significantly different if the *p*-value was less than 0.05, a commonly accepted threshold in statistical hypothesis testing. In this case, we obtained a *p*-value of 0.0001, indicating a highly significant difference between the two groups. This low *p*-value suggests that, assuming the null hypothesis is true, the likelihood of obtaining results as extreme as ours due to random variation is only 0.01%. The means of the two groups (2.477 ± 0.11 and 0.5967 ± 0.06) exhibit a substantial difference, further supporting the conclusion of a meaningful effect. These findings were further confirmed by immunoblotting analysis of hippocampal samples, which showed a significant increase in the gauche P-tau conformer in AD brains (1.38 ± 0.35 vs. 0.38 ± 0.13, *n* = 5, *p* = 0.031) (Figure 1c). However, no significant differences in cis P-tau levels were observed between AD and control brains (0.208 ± 0.1 vs. 0.5 ± 0.09, *n* = 5, *p* = 0.068), nor were there significant differences in trans P-tau and tau5 levels (0.05008 ± 0.01 vs. 0.05429 ± 0.002, *n* = 5, *p* = 0.722; 0.1003 ± 0.04 vs. 0.2611 ± 0.11, *n* = 5, *p* = 0.244) (Figure 1d).

In summary, our findings demonstrate a significant increase in gauche P-tau levels in the AD brain. In contrast, no significant differences were observed in cis and trans P-tau levels between the AD and control groups. Additionally, we performed co-staining analysis with cis/gauche as well as trans/gauche p-tau to examine any colocalization. Quantitative co-localization analysis using Pearson’s correlation coefficient showed minimal overlap between gauche and cis/trans conformers, supporting their distinct identities (Supplementary Figure S6).

### 3.4. Cis P-Tau Accumulation Was Observed in Mouse Primary Cortical Neurons Under Aging Stress

**Figure 2:**
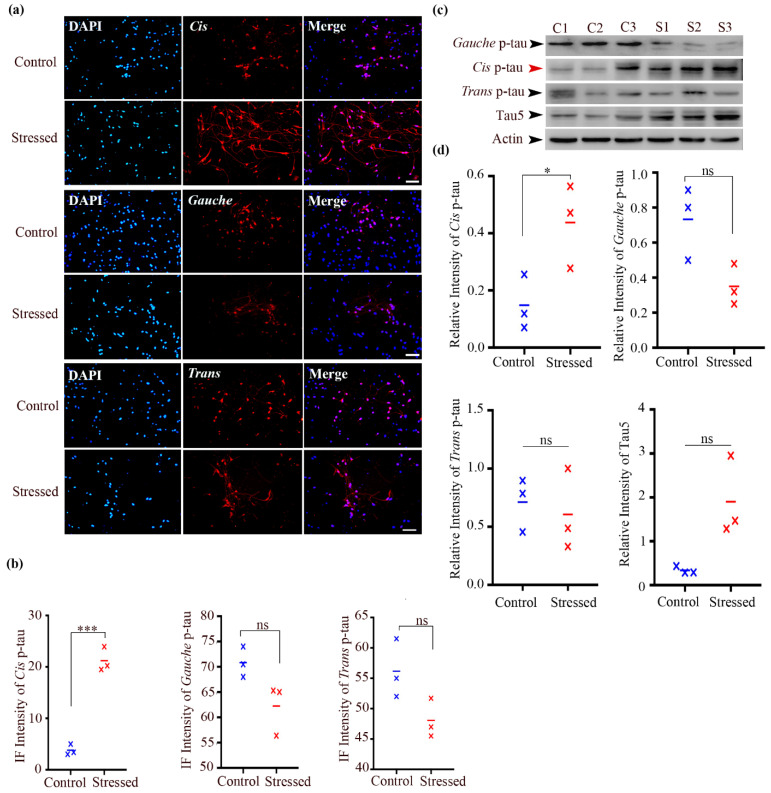
Aging stress induces a more profound accumulation of cis P-tau compared to gauche P-tau in mouse cortical neurons. (**a**) Immunofluorescent staining of neurons using monoclonal antibodies against cis, trans, and gauche P-tau (Cis, red; Trans, red; Gauche, red). DNA was counterstained with DAPI (blue). Control condition corresponds to DIV 0. Scale bar, 100 μm. (**b**) Quantification of cis, trans, and gauche P-tau levels in control and aging stress-treated groups. Data are presented as mean ± SEM; *n* = 3 independent cell culture preparations per group; n.s.: not significant; *** *p* < 0.001 by unpaired *t*-test. (**c**) Immunoblot analysis of mouse cortical neurons under aging stress, probed with tau5, cis, trans, and gauche P-tau monoclonal antibodies. (**d**) Quantification of immunoblots from panel c. Data are presented as mean ± SEM; *n* = 3 independent cell culture preparations per group; n.s.: not significant; * *p* < 0.05 by unpaired t-test. Original Western blot images can be found in Figure S8.

Embryonic primary cortical neurons were generated to explore the cellular response to aging stress (Supplementary Figure S5). Immunofluorescence staining was performed using anti-cis, trans, and gauche P-tau monoclonal antibodies (mAbs) on cultured neurons under aging stress conditions. Our findings indicated a significant increase in cis P-tau levels in stressed neurons compared to the control samples (21.22 ± 1.37 vs. 3.8 ± 0.611, *n* = 3, *p* = 0.0003). However, no significant alterations were observed in trans and gauche P-tau levels under aging stress in comparison to the control conditions (48.07 ± 1.8 vs. 56.17 ± 2.8, *n* = 3, *p* = 0.074 and 62.23 ± 2.92 vs. 70.83 ± 1.74, *n* = 3, *p* = 0.0674, respectively, Figure 2a,b).

Furthermore, immunoblotting analysis confirmed a robust increase in cis P-tau in response to aging stress (0.4377 ± 0.08 vs. 0.1485 ± 0.05, *n* = 3, *p* = 0.046). This induction correlated with overall tau levels in the neurons (1.904 ± 0.52 vs. 0.3367 ± 0.04, *n* = 3, *p* = 0.042, Figure 2c,d). Additionally, the immunoblotting results showed no significant changes in trans and gauche P-tau levels in neurons under aging stress (0.6063 ± 0.2 vs. 0.713 ± 0.13, *n* = 3, *p* = 0.682 and 0.35 ± 0.06 vs. 0.73 ± 0.12, *n* = 3, *p* = 0.05, respectively). In summary, our observations confirmed the induction of cis P-tau in mouse neurons in response to neuronal stress, consistent with prior findings.

### 3.5. There Was a Profound Gauche pT231-Tau Accumulation in Cultured Human Neurons upon Aging Stress

In this study, we explored the accumulation of tau conformers—cis, trans, and gauche P-tau—in human neurons subjected to aging stress. Neurons derived from the AD3 hiPSC line were used to assess the cellular response to aging. In human postmortem AD brains, we observed elevated gauche pT231-tau levels, which were also present in iPSC-derived neurons under aging-related stress (74.67 ± 8.66 vs. 20.27 ± 2.80, *n* = 3, *p* = 0.0039). This supports previous findings indicating the potential pathogenic role of gauche P-tau in tauopathies.

There was also an increase in cis P-tau in aging-stressed neurons (37.67 ± 1.45 vs. 29 ± 2, *n* = 3, *p* = 0.0248). Although this accumulation was less pronounced than that of gauche P-tau, it still suggests a shift in tau conformation under aging stress, likely reflecting its pathogenic nature.

**Figure 3:**
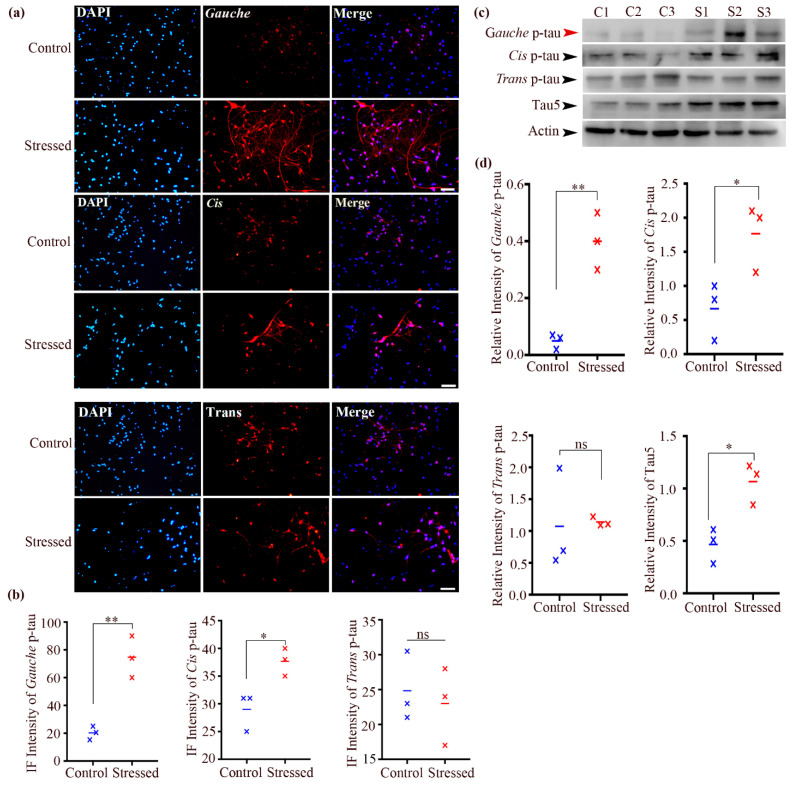
Aging stress induces profound accumulation of gauche P-tau in human neurons. (**a**) Immunofluorescent staining of human neurons using monoclonal antibodies against cis, trans, and gauche P-tau. Scale bar, 100 μm. (**b**) Quantification of cis, trans, and gauche P-tau positive cells under aging stress. Data are presented as mean ± SEM; *n* = 3, independent cell culture preparations per group, normalized to equal density; n.s.: not significant; * *p* < 0.05 and ** *p* < 0.01 by unpaired *t*-test. (**c**) Neurons cultured under aging stress were analyzed by immunoblotting for Tau5, cis, trans, and gauche P-tau levels. (**d**) Quantification of immunoblot data from part c. Data are presented as mean ± SEM; *n* = 3, independent cell culture preparations per group, normalized to equal density; n.s.: not significant; * *p* < 0.05, ** *p* < 0.01 by unpaired *t*-test. Original Western blot images can be found in Figure S8.

Trans P-tau levels remained unchanged under aging stress (23 ± 3.21 vs. 24.83 ± 2.8, *n* = 3, *p* = 0.693), suggesting that this conformer may not be significantly affected by aging stress (Figure 3a,b).

Immunoblotting confirmed the significant increase in gauche P-tau levels due to aging stress (0.4 ± 0.05 vs. 0.05 ± 0.01, *n* = 3, *p* = 0.004). This induction was found to correlate with the overall tau levels observed in the neurons (1.065 ± 0.11 vs. 0.4667 ± 0.09, *n* = 3, *p* = 0.016).

The cis P-tau levels also increased (1.767 ± 0.28 vs. 0.66 ± 0.24, *n* = 3, *p* = 0.042), consistent with the immunostaining results.

No significant change was observed in trans P-tau levels (1.142 ± 0.04 vs. 1.074 ± 0.45, *n* = 3, *p* = 0.889), reinforcing the notion that trans P-tau may not play a major role under these conditions (Figure 3c,d).

These findings highlight that gauche P-tau is the most notably accumulated tau conformer in response to aging stress, with cis P-tau showing some increase as well. In contrast, trans P-tau levels remained stable. This suggests that gauche P-tau may play a more significant role in tauopathies associated with aging. Taken together, we found that while aging stress induces profound cis p-tau accumulation in mouse cultured neurons, human AD neurons exhibit more pronounced gauche p-tau accumulation. We observed a significant increase in gauche pT231-tau levels in the AD brain, while cis and trans pT231-tau levels remained unchanged between the AD and control groups. In contrast, in human AD cultured neurons, we found an increase in cis pT231-tau. This discrepancy may be due to the different timelines for tau pathology between cultured neurons (45 days) and human AD brains (which undergo tau pathology over several years). The accelerated disease progression in the in vitro model could lead to different patterns of tau accumulation compared to the chronic and complex tau pathology in postmortem human brains.

### 3.6. Distinct Functionalities: Gauche P-Tau Functions Independently of Cis P-Tau

While tau misfolding and aggregation are key drivers of neurodegeneration in AD, we found that the cis pT231-tau conformer was significantly increased in mouse neurons, whereas the gauche pT231-tau conformer was specifically elevated in human neurons. These findings suggest species-specific differences in tau pathology in neurodegeneration.

**Figure 4:**
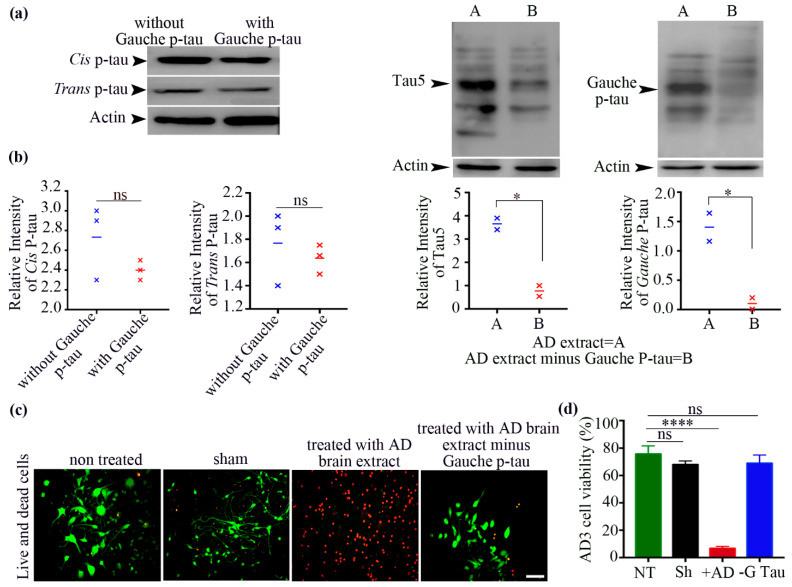
Accumulation of gauche p-Tau in human AD brains is neurotoxic. (**a**) Immunoblot analysis of AD brain extract and gauche p-Tau-immunodepleted extract detecting cis and trans p-Tau. (**b**) Immunoblot analysis of AD brain extract and gauche p-Tau-immunodepleted extract detecting gauche p-Tau and total Tau (Tau5). Data are presented as mean ± SEM; *n* = 3 participants; n.s.: not significant; * *p* < 0.05 by unpaired *t*-test. (**c**) Human neurons at day 27 in vitro were treated with AD brain extract or AD extract depleted of gauche p-Tau, followed by live/dead cell assays. Green: live cells; red: dead cells. Scale bar: 100 µm. (**d**) Quantitative analysis of results shown in part c. (**** *p* < 0.0001, n.s., not significant; one-way ANOVA; *n* = 3 independent cell culture preparations per group at equal density; mean ± SEM). NT: non-treated, Sh: sham, +AD: treated with AD brain extract, -G Tau: treated with AD brain extract minus gauche p-tau. Original Western blot images can be found in Figure S8.

To further validate the existence of gauche p-tau and distinguish it from other p-tau conformers, we immunodepleted gauche p-tau from Alzheimer’s disease (AD) brain extracts and assessed the remaining tau population using a pan-tau antibody (Tau-5). Western blot analysis demonstrated that depletion of gauche p-tau did not alter the levels of cis or trans p-tau, as detected by their respective conformer-specific mAbs (Figure 4a). However, total tau levels were significantly reduced (~80%), indicating that the majority of tau in AD brain extracts exists in the gauche conformation rather than in the cis or trans forms (Figure 4b).

Brain lysates were prepared from Alzheimer’s disease (AD) brain extracts, either untreated or depleted of gauche P-tau, and added to cultured neurons on day 27 for a three-day assessment of gauche P-tau’s neurotoxic effects. Neurons treated with AD extracts showed significantly higher rates of cell death compared to those treated with gauche P-tau-depleted extracts, untreated controls, or sham-treated controls. Notably, the addition of AD brain extract, but not the gauche P-tau-depleted extract, induced neuronal cell death (Figure 4c,d). Neurons were exposed to AD brain extracts for 48 h. While significant cell death was observed, residual viable neurons were still detectable, indicating partial but not complete toxicity.

While cis p-tau has been reported as neurotoxic, in our experimental setup, we did not observe significant pathogenic effects from either cis or trans p-tau. This could be due to the low amounts of these species present in the immunodepleted samples, which may have been insufficient to elicit detectable toxicity in our cell culture system.

### 3.7. Elimination of Cis P-Tau Using the Respective mAb Diminished Neurodegeneration in Stressed Primary Cultured Mouse Neurons

**Figure 5:**
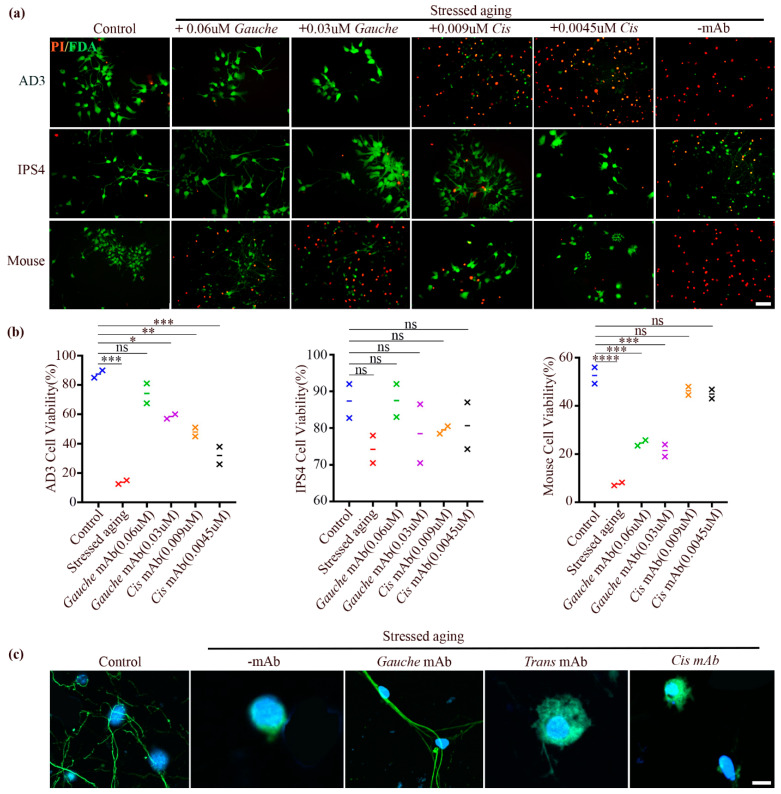
Neuronal aging stress induced the accumulation of gauche p-tau and cis p-tau, along with microtubule disruption in human and mouse cortical neurons, respectively. These effects were blocked by their respective mAbs. (**a**) Human AD3, IPS4, and primary mouse cortical neurons were subjected to aging stress in the absence or presence of cis and gauche p-tau mAbs, followed by a live/dead cell assay (live cells in green; dead cells in red). (**b**) Quantitative analysis of results shown in part A. (**** *p* < 0.0001, *** *p* < 0.001, ** *p* < 0.01, * *p* < 0.05; n.s., not significant; one-way ANOVA; *n* = 3 independent cell culture preparations per group at equal density; mean ± SEM). Scale bars, 100 μm. (**c**) Immunofluorescence staining of microtubules in human neurons subjected to aging stress in the absence or presence of cis, trans, and gauche p-tau mAbs. Scale bars, 20 μm. AD3, cells derived from AD human; IPS4, cells derived from healthy human.

It has been previously reported that nutritional starvation or hypoxia stress triggers the emergence of cis p-tau in primary mouse neurons. Importantly, the elimination of cis p-tau through passive immunotherapy effectively diminished tau pathogenicity and neurodegeneration in these cultured neurons [3,17,48,49]. Here, to examine the impact of aging stress on the viability of mouse cortical neurons, a live/dead cell assay was performed. Under stress conditions, neurodegeneration was observed, whereas the application of the cis p-tau mAb not only reduced cis p-tau levels but also diminished cell death, as shown in Figure 5a. Quantification revealed that the mean viability rates significantly improved to 46.25 ± 1.75 and 44.9 ± 1.9 in cells treated with 0.009 and 0.0045 μM cis p-tau mAb, respectively. In contrast, viability rates were 24.65 ± 1.15 and 21.5 ± 2.5 in cells treated with 0.06 and 0.03 μM of the gauche p-tau mAb, as depicted in Figure 5a,b. Various mAb concentrations were tested, and the dosages employed in this study were selected based on these evaluations. In fact, the applied antibodies have different Kd values (Supplementary Figure S7), resulting in varying binding affinities and efficiencies, which are reflected in their effective concentrations.

### 3.8. Elimination of Gauche pT231-Tau Using Its Respective mAb Suppressed Microtubule Disruption and Neurodegeneration in Stressed Cultured Human Neurons

**Figure 6:**
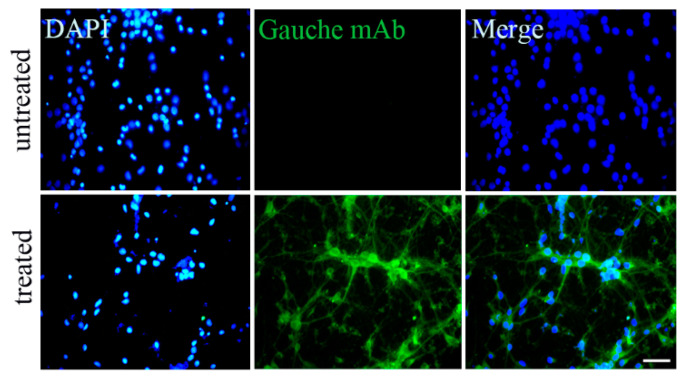
Internalization of gauche mAbs in neurons. Neurons were treated with gauche monoclonal antibodies (mAbs), followed by immunofluorescence staining to assess antibody penetration into the cells. Scale bar: 50 µm.

To better understand the role of tau protein conformations in Alzheimer’s disease development in mice and humans, passive immunotherapy was applied to eliminate pathogenic conformers. As mentioned above, the elimination of cis p-tau using the cis p-tau mAb was confirmed to halt neurodegeneration in stressed cultured mouse neurons. Next, we examined whether immunodepletion using cis, gauche, and trans p-tau mAbs could rescue cell death in human neurons (Figure 5a,b). Additionally, we investigated whether gauche p-tau mAb could enter neurons to facilitate targeted protein degradation. Gauche mAb was confirmed to enter neurons and promote the breakdown of gauche p-tau (Figure 6).

We found that, while cis or trans p-tau mAbs did not suppress neurodegeneration in the human AD3 cell line, treatment with 0.06 and 0.03 μM gauche p-tau mAb increased cell viability to 74.26 ± 6.74 and 58.5 ± 1.5, respectively, compared to the stressed control group (13.75 ± 1.25). In contrast, treatment with 0.009 and 0.0045 μM cis p-tau mAb resulted in negligible viability improvements (13.75 ± 3 and 13.75 ± 6, respectively). Similar trends were observed in the human IPS4 cell line, where treatment with 0.06 μM and 0.03 μM gauche p-tau mAb improved cell viability to 87.5% ± 4.5% and 78.5% ± 8.0%, respectively, compared to 79.5% ± 1.0% and 80.65% ± 6.35% for cells treated with 0.009 μM and 0.0045 μM cis p-tau mAb (Figure 5a,b).

Furthermore, we investigated the microtubule network in the human AD3 cell line under aging stress and assessed the effects of mAb treatment. Aging stress induced gauche p-tau accumulation without concurrent accumulation of trans or cis p-tau, leading to axonal microtubule disruption. Importantly, treatment with an anti-gauche p-tau mAb restored the microtubule structure (Figure 5c).

Overall, these findings indicate that the gauche p-tau conformer is induced under aging stress and can be identified as a neurotoxic factor in human neurons. Furthermore, treatment with anti-gauche p-tau mAb effectively mitigates the toxicity associated with the presence of the gauche p-tau conformer in cells subjected to aging stress.

### 3.9. The Structure of the Tau Protein Differs Between Humans and Rodents

Given the differences between human and mouse neurons [50,51,52,53], it is reasonable to assume that tau proteins exhibit variations in both structure and function across these species. Despite their high sequence similarity, human and mouse tau proteins differ by 11 amino acids at the N-terminal region, a key determinant of their functional differences [50]. Additionally, there are small yet functionally significant variations in three amino acid residues between human and mouse tau (S238A, A239S, and K257R) [54]. One notable difference is the reversal of the serine-alanine (S238A239) arrangement in human tau compared to mouse tau. In human tau, serine 238 (S238) forms a transient salt bridge with arginine 242 (R242) [55], which is absent in mouse tau. The displacement of alanine and serine in mouse tau prevents salt bridge formation due to unfavorable distances. This salt bridge in human tau likely contributes to increased microtubule protein dynamics in the human brain.

**Figure 7:**
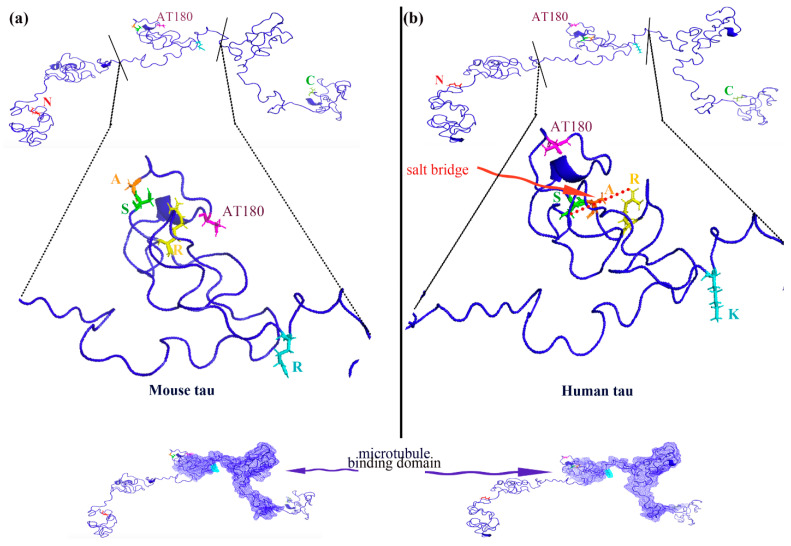
Three-dimensional models of tau protein were generated using PYMOL version 1.20, with structures built by i-TASSER. (**a**) Protein model superimposition of mouse tau (*p*-score = 0.38). (**b**) Protein model superimposition of human tau (*p*-score = 0.35).

Consequently, these sequential differences have a significant impact on tau conformation and function (Figure 7a,b). Considering these insights, the variations in tau primary structure between humans and mice offer valuable context for understanding the toxicity of the novel pT231-tau conformer in human AD neurons, a notion supported by our experimental findings.

## 4. Discussion

Tau protein exists in multiple conformations, each with distinct structures that lead to diverse biological functions. Due to its conformational diversity, tau is involved in a range of roles [55,56,57], including regulating microtubule dynamics and protecting microtubules from depolymerization [58]. The pathogenicity of tau is a crucial step in the progression of neurodegenerative diseases, such as Alzheimer’s disease (AD). Previous studies have highlighted the role of cis pT231-tau as a central mediator of tauopathy and neurodegeneration in traumatic brain injury (TBI) mouse models [17]. Conformation-specific antibodies targeting either cis or trans pT231-tau have been recognized [25], and notably, anti-cis p-tau mAb has been shown to block tau pathology and neurodegeneration in TBI mouse models [17,59]. Tau phosphorylation at T231 is critical for tau pathology [21,60]. However, we observed slight differences between human and rodent tau proteins in the AT180 domain, which led us to speculate the existence of another pT231-tau conformer distinct from cis or trans forms. This conformer may contribute to tau pathogenicity in the human nervous system [34].

Our objective in this study was to identify which pT231-tau conformer accumulates in human AD brains. To achieve this, immunohistochemical staining was performed on postmortem brain sections from both AD patients and healthy controls, using anti-cis, trans, and gauche pT231-tau antibodies. Our findings revealed that gauche pT231-tau significantly accumulated in AD brains compared to healthy controls, suggesting its potential role in tau pathogenicity in human neurons, as indicated by both in vitro and in vivo AD models. Unlike previous studies focused on cis pT231-tau [26], gauche pT231-tau was observed in both postmortem and in vitro models under aging stress, suggesting a unique pathogenic mechanism.

While some alterations in trans and cis p-tau levels were observed in human AD neurons, our study highlighted the pivotal role of gauche p-tau in mediating neurodegeneration in the human AD brain, a finding supported by observations in neurons derived from AD iPSC cell lines. Live/dead cell assays conducted on both mouse cortical neurons and human iPSC-derived neurons under aging-induced stress demonstrated that elimination of cis p-tau using a conformation-specific antibody halted neurodegeneration in mouse primary cortical neurons. More importantly, our newly developed anti-gauche p-tau mAb significantly reduced tau pathogenicity and neurodegeneration in human AD neurons.

This study also demonstrated that the gauche pT231-tau conformer induces microtubule instability and tau protein oligomerization, which warrants further investigation. While maturation times can vary between cultures, the consistent accumulation of p21 and tau misfolding across replicates indicates that these differences are not attributable to culture variability. This consistency supports the reliability of our findings despite the inherent variability in primary neuronal cultures. Therefore, we propose that the newly identified pT231-tau conformer, distinct from both cis and trans forms, likely accumulates in the human AD brain, contributing to tau pathogenicity.

Although we have not definitively confirmed that the newly found p-tau conformer is the gauche conformer, the fact that it differs from both cis and trans conformations suggests this possibility. Although crystallographic validation remains pending, the structural model aligns with known tau folding patterns based on molecular dynamics simulations. Additional studies are needed to characterize the exact conformation of this newly introduced pT231-tau conformer.

Tau hyperphosphorylation at the AT180 domain is an established event in tau pathogenicity. Our findings indicate moderate alterations in cis and trans pT231-tau conformations during AD progression in the human nervous system. Notably, the novel p-tau conformer accumulates in AD brains, potentially leading to neurodegeneration as the disease develops. Taken together, these findings suggest that the newly identified p-tau conformer could serve as an effective therapeutic target for combating Alzheimer’s disease.

## 5. Conclusions

Our study identifies a novel gauche pT231-tau conformer, distinct from previously characterized cis and trans pT231-tau, as a key pathogenic factor in human Alzheimer’s disease (AD) neurons. This conformer was found to accumulate specifically in AD brains and aged human neurons, contributing to neuronal toxicity and microtubule disruption. Notably, targeted immunotherapy against gauche pT231-tau mitigated neurodegeneration, highlighting its potential as a therapeutic target for AD. These findings provide new insights into tau-mediated neurodegeneration in human neurons, differentiating it from tauopathies observed in rodent models. Future studies should explore structural validation of gauche pT231-tau, its role in tau propagation, and its therapeutic targeting in clinical settings.

## Figures and Tables

**Table 1:** Primary antibodies used.

Antibody	Immunogen	Company Name, Source Cat#, RRID Host, Clonality	Concentration Used
Conformation specific *cis* P-Thr231tau	Synthetic peptide corresponding to the pThr231 tau in cis conformation	Gift Prof. KP Lu Cat# KPLu_2012, RRID: AB_2877630 Mouse monoclonal	IF: 1/500 Wb: 1/2500
Conformation specific *trans* P-Thr231tau	Synthetic peptide corresponding to the pThr231 tau in *trans* conformation	Gift Prof. KP Lu Cat# KPLu_2012–2 RRID: AB_2877667 Mouse monoclonal	IF: 1/500 Wb: 1/2000
Conformation-independent P-Thr231 tau	Synthetic peptide corresponding to the pThr231 tau in a conformation different from either cis or trans	Patent No.: US 10,570,195 B2 [34]	IF: 1/500 Wb: 1/1500
Tau-5	Tau antibody, raised against a.a. 210–241, clone Tau-5	Millipore, Cat# MAB361, RRID: AB_94944 Mouse monoclonal	Wb: 1/1000
Anti-Tubulin β 3 antibody	Raised against Tubulin beta-3 chain derived from rat brain	BioLegend, Cat# 801213, RRID: AB_2313773 Mouse monoclonal	IF: 1/1000
Anti-β Actin antibody	Raised against actin, cytoplasmic 1	Proteintech, Cat# IG-60008-1, RRID: AB_2289225 Mouse monoclonal	Wb: 1/30,000
Anti-MAP-2 Antibody	Raised against microtubule-associated protein 2	MilliporeSigma, Cat# M1406, RRID: AB_477171 Mouse monoclonal	IF: 1/500
Anti-GABA Antibody	Raised against gamma-aminobutyric acid	MilliporeSigma, Cat# AB131, *RRID*: AB_2278931 Rabbit Antibody	IF: 1/500
Anti-p21 Antibody	Raised against cyclin-dependent kinase inhibitor 1	*Abcam*, UK, Cat# ab16767, Mouse monoclonal	IF: 1/200

**Table 2:** The clinical characteristic of human subjects.

Cases	Age	Gender	Braak Stage	Diagnostic Criteria
Control				
1	71	female	N/A	Clinical history
2	67	female	N/A	Clinical history
3	65	male	N/A	Clinical history
4	61	male	N/A	Clinical history
5	77	female	N/A	Clinical history
AD				
1	65	male	AD stage 4	Clinical history
2	68	female	AD stage 3	Clinical history
3	70	male	AD stage 4	Clinical history
4	66	male	AD stage 4	Clinical history
5	65	female	AD stage 3	Clinical history

## Data Availability

The datasets generated and analyzed in this study are available from the corresponding author upon reasonable request.

## Supplementary Materials

The following supporting information can be downloaded at https://www.mdpi.com/article/10.3390/biom15040585/s1: Figure S1: hiPSC differentiation into mixed cortical neurons; Figure S2: Immunofluorescence Staining of NPCs (a) and Neurons (b) Derived from hiPSCs; Figure S3: Human iPSC-derived neurons under aging stress stained for P21; Figure S4: Mouse cortical neurons stained for P21; Figure S5: Schematic representation of long-term primary neuronal culture from embryonic mouse cortex. Dissociated neurons were plated onto coverslips at Day 0 in vitro (DIV 0). After approximately 7 DIV, and following long-term culture, the neurons begin to acquire senescent features. DIV refers to days in vitro; Figure S6: Co-immunostaining for cis, trans, and gauche pT231-tau in postmortem AD brains. Quantitative co-localization analysis using Pearson’s correlation coefficient showed minimal overlap between gauche and cis/trans conformers, supporting their distinct identities: Pearson’s _Rr = 0.0707156 for cis/gauche co-stain. Pearson’s _Rr = 0.6537089 for trans–gauche co-stain; Figure S7: We compared antibody affinities through ELISA-based titrations using synthetic peptides for cis, trans, and gauche pT231-tau. While all three antibodies demonstrated high specificity, their binding affinities differed slightly; Figure S8: Original Western blot images; Table S1: Supplemental table containing the raw intensity values and statistical details for key blots. Table S2: Supplementary table with the titration data, which includes the various mAb concentrations tested and the corresponding dosages selected for the study.

## Abbreviations

AD, Alzheimer’s disease; NFTs, neurofibrillary tangles; Pin1, peptidyl prolyl isomerase 1; TBI, traumatic brain injury; hiPSCs, human-induced pluripotent stem cells; DMEM, Dulbecco’s modified Eagle’s medium; KOSR, knockout serum; NEAAs, non-essential amino acids; ITS, insulin transferrin selenium; bFGF, basic fibroblast growth factor; BDNF, brain-derived neurotrophic factor; GDNF, glial cell line-derived neurotrophic factor; hiPSC-NPs, human-induced pluripotent stem cell-derived neural progenitors; FDA, fluorescein diacetate; PI, propidium iodide; NPCs, neural progenitor cells; mAbs, monoclonal antibodies; BCA, bicinchoninic acid; PVDF, polyvinylidene fluoride; HRP, horseradish peroxidase; ECL, enhanced chemiluminescence; DAPI, 4,6-diamidino-2-phenylindole; I-TASSER, iterative threading assembly refinement; ANOVA, analysis of variance; DDR, DNA damage response; CTE, chronic traumatic encephalopathy; S238, serine 238; R242, arginine 242; S238A, serine238alanine; K257R, lysine257arginine; AT180, phosphorylated Thr231-tau; cis P-tau, *Cis* phosphorylated Thr231-tau; gauche P-tau, *gauche* phosphorylated Thr231-tau; trans P-tau, *trans* phosphorylated Thr231-tau; Thr, threonine; Pro, proline; GABA, gamma-aminobutyric acid; GAD, glutamic acid decarboxylase; MAP2, microtubule-associated protein 2; SOX2, SRY-box transcription factor 2; PAX6, paired box 6; Nestin, neuroepithelial stem cell protein.
