# Supplementary material for: A Novel Phosphorylated Tau Conformer Implicated in the Tauopathy Pathogenesis of Human Neurons
Source: Biomolecules. 2025 Apr 15;15(4):585. doi: 10.3390/biom15040585 (PMC12025006; doi:10.3390/biom15040585)
Supplement: Supplementary file 1 [file biomolecules-15-00585-s001.zip › biomolecules-3509752-supplementary.pdf]

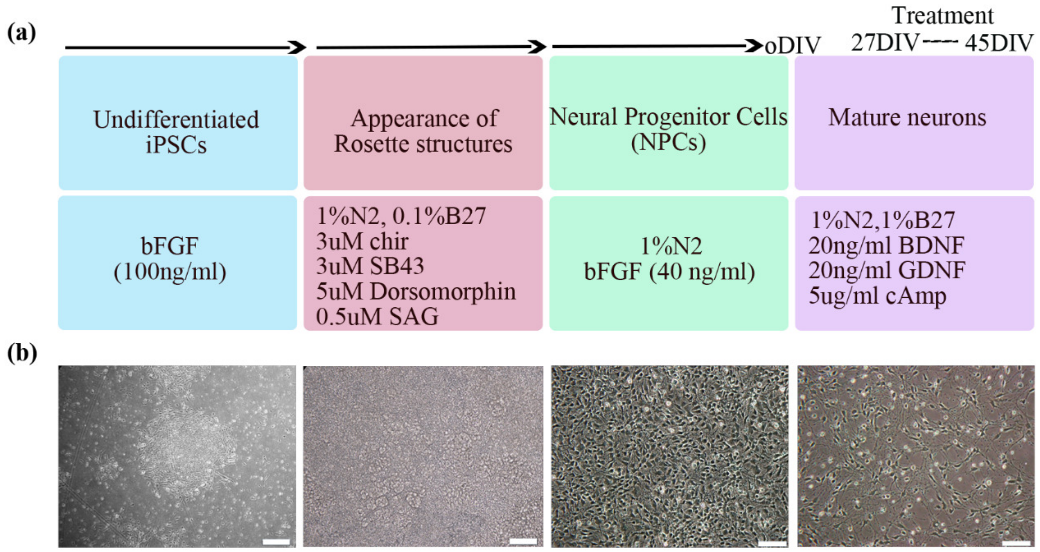

Figure S1. hiPSC Differentiation into Mixed Cortical Neurons. (a) Schematic representation of the differentiation protocol. (b) Representative phase-contrast images showing the progression of hiPSC differentiation. From left to right: hiPSC colonies (scale bar = 500 µm), neural rosettes (scale bar = 100 µm), rosette-derived human neural progenitor cells (hNPCs) (scale bar = 200 µm), and neuronal cells (scale bar = 100 µm).

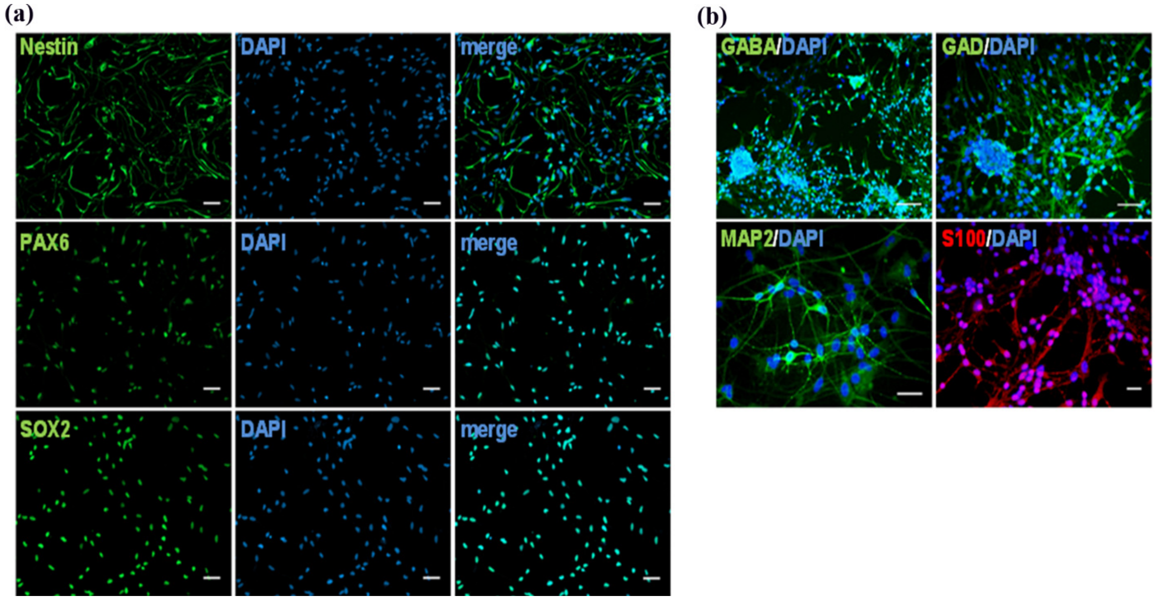

Figure S2. Immunofluorescence Staining of NPCs (a) and Neurons (b) Derived from hiPSCs. NPCs were generated from hiPSCs and subsequently induced to differentiate into neurons. Immunofluorescence staining was performed for NPC markers Nestin, PAX6,

and SOX2 (scale bars, 100 µm), as well as mature neuronal markers GABA, GAD, and MAP2 (scale bars, 100 µm for GABA, GAD; scale bar, 50 µm for MAP2). NPC, neural progenitor cell; hiPSC, human induced pluripotent stem cell.

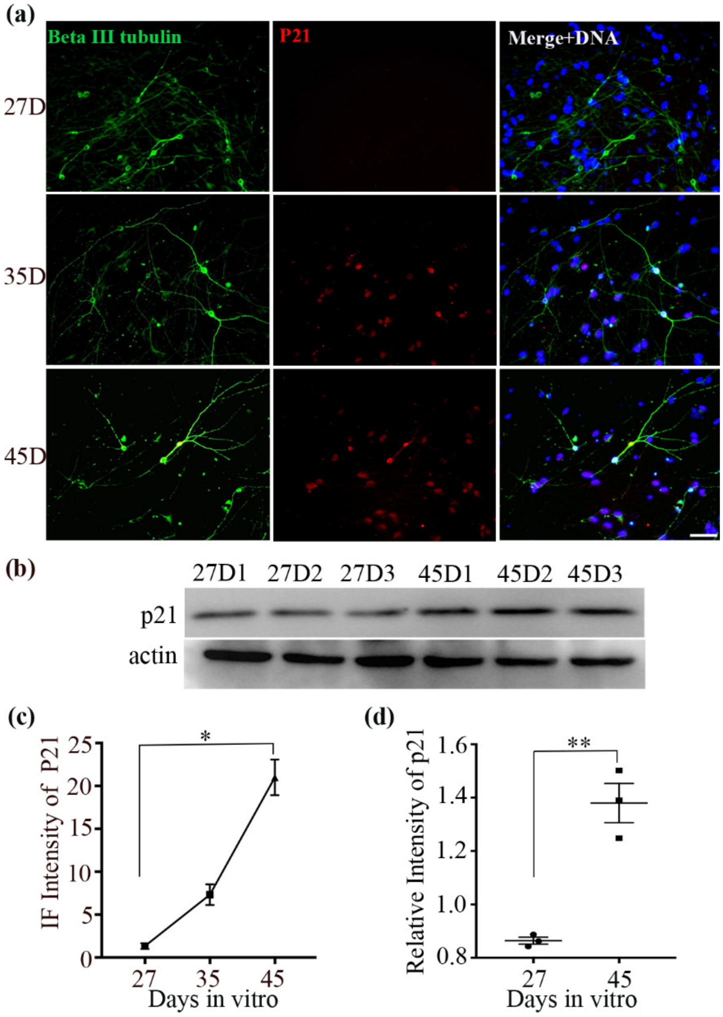

Figure S3. Human iPSC-derived neurons under aging stress stained for P21. (a) Immunofluorescence staining of neurons expressing βIII-TUBULIN, incubated for the indicated number of days in vitro. Scale bar, 100 µm. (b) Immunoblotting of neurons at day 27 (control) and day 45 (aged). (c) Quantification of p21 levels in neurons. Data are presented as mean ± SEM; n = 3 independent cell culture preparations per group, normalized to equal density. *$p < 0.05$, one-way ANOVA with Tukey's multiple comparison test. (d) Quantification of immunoblot data from panel (b). Data are mean ± SEM; **$p < 0.01$, unpaired t-test; n = 3 independent cell culture preparations per group.

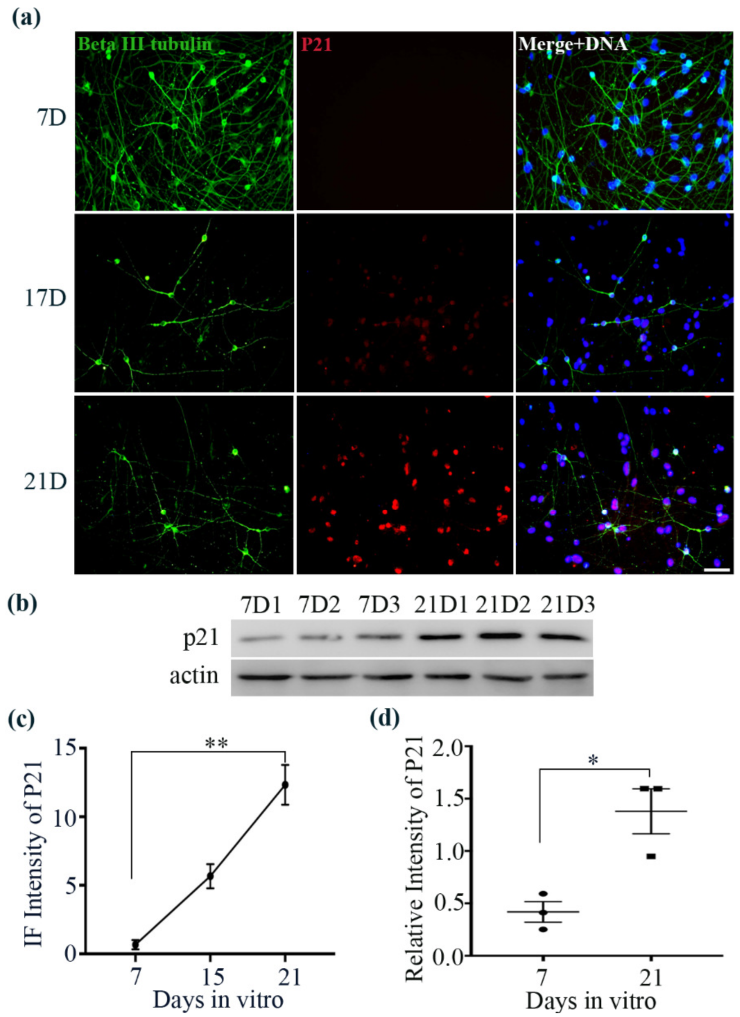

Figure S4. Mouse cortical neurons stained for P21. (a) Immunofluorescence staining of neurons expressing βIII-TUBULIN, incubated for the indicated number of days in vitro. Scale bar, 100 μm.

(b) Immunoblotting of neurons at day 7 (control) and day 21 (stressed). (c) Quantification of p21 levels in neurons. Data are presented as mean ± SEM; n = 3 independent cell culture preparations per group, normalized to equal density. $p < 0.01$, one-way ANOVA with Tukey's multiple comparison test. (d) Quantification of immunoblot data from panel (b). Data are mean ± SEM; *$p < 0.05$, unpaired t-test; n = 3 independent cell culture preparations per group.

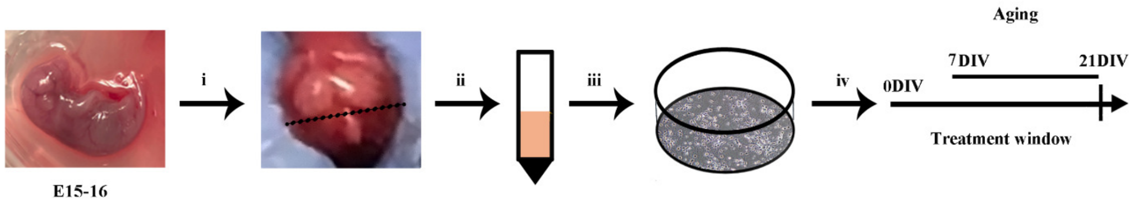

Figure S5. Schematic representation of long-term primary neuronal culture from embryonic mouse cortex. Dissociated neurons were plated onto coverslips at Day 0 in vitro (DIV 0). After approximately 7 DIV, and following long-term culture, the neurons begin to acquire senescent features. DIV refers to days in vitro.

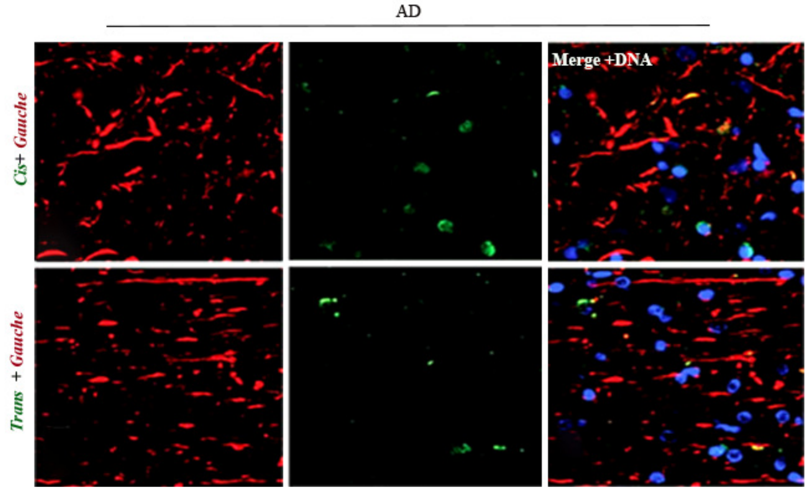

Figure S6: Co-immunostaining for cis, trans, and gauche pT231-tau in postmortem AD brains. Quantitative co-localization analysis using Pearson's correlation coefficient showed minimal overlap between gauche and cis/trans conformers, supporting their distinct identities:

Pearson's _Rr = 0.0707156 for cis/gauche co-stain

Pearson's _Rr = 0.6537089 for trans-gauche co-stain

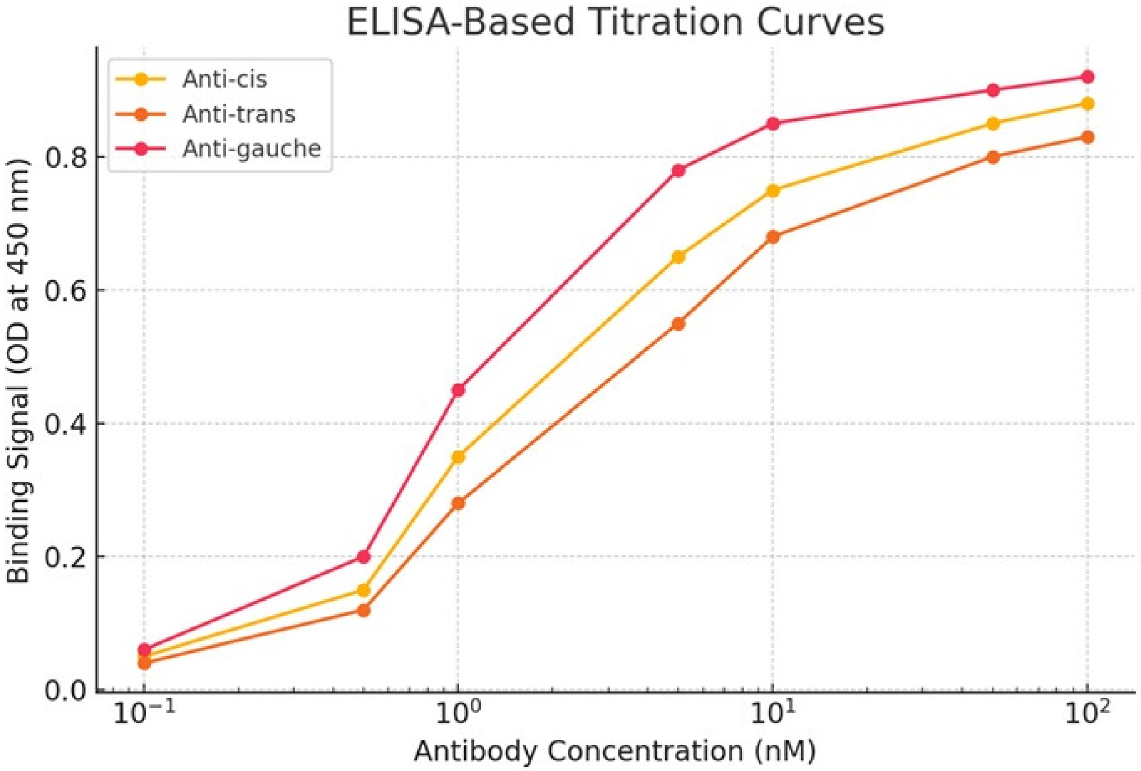

Figure S7: We compared antibody affinities through ELISA-based titrations using synthetic peptides for cis, trans, and gauche pT231-tau. While all three antibodies demonstrated high specificity, their binding affinities differed slightly.

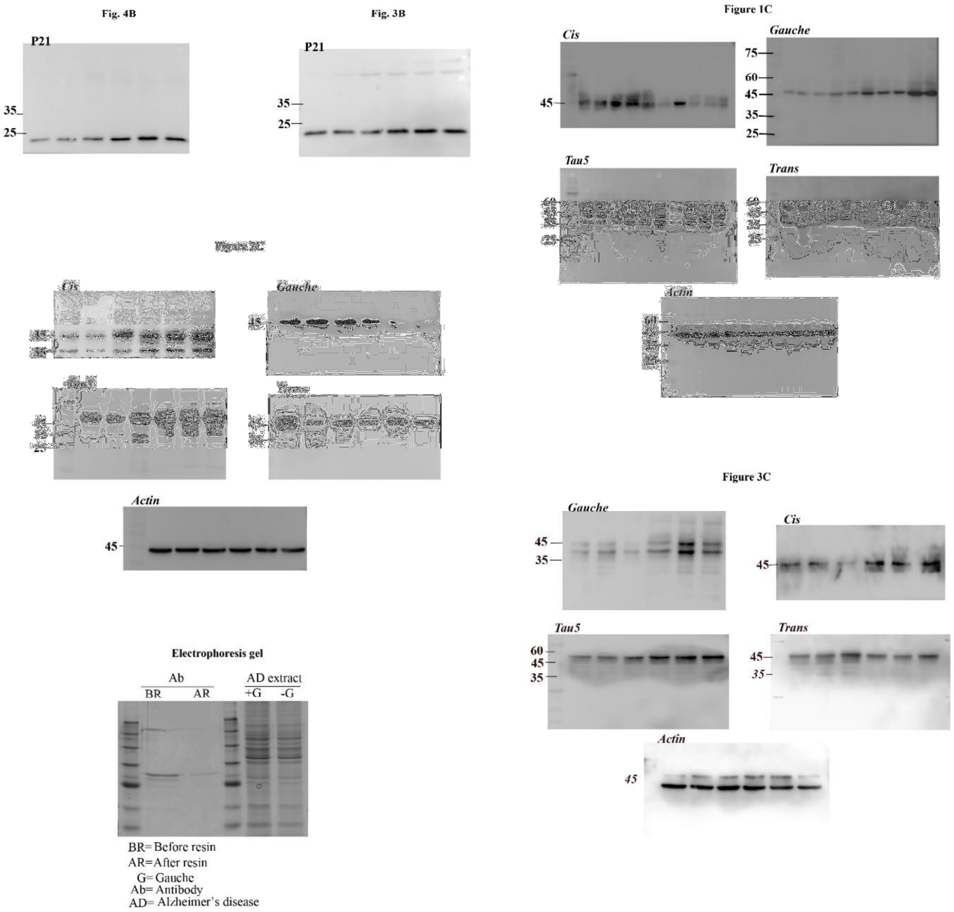

Figure S8: Original Western blot images.

Table S1: Supplemental table containing the raw intensity values and statistical details for key blots

Assay	Name of P-tau	mean ± SEM Control	mean ± SEM AD	P value	Test description
**P-tau intensity**	Cis P-tau	0.5 ± 0.09	0.208 ± 0.1	p = 0.068	unpaired t-test
	Trans P-tau	0.05429 ± 0.002	0.05008 ± 0.01	p = 0.722	unpaired t-test
	Gauche P-tau	0.38 ± 0.13	1.38 ± 0.35	p = 0.031	unpaired t-test
**P-tau positive cells**	Cis P-tau	2.393 ± 0.21	1.597 ± 0.06	p = 0.0233	unpaired t-test

	Trans P-tau	1.293 ± 0.109	1.407 ± 0.209	p = 0.6572	unpaired t-test
	Gauche P-tau	0.5967 ± 0.06	2.477 ± 0.11	p = 0.0001	unpaired t-test

Note: unpaired t-test Cis P-tau, Trans P-tau, and Gauche P-tau (Figure 1)

**Assay**	**Name of P-tau**	**mean ± SEM Control**	**mean ± SEM aging stress**	**P value**	**Test description**
**P-tau intensity**	Cis P-tau	0.1485 ± 0.05	0.4377 ± 0.08	p = 0.046	unpaired t-test
	Trans P-tau	0.713 ± 0.13	0.6063 ± 0.2	p = 0.682	unpaired t-test
	Gauche P-tau	0.73 ± 0.12	0.35 ± 0.06	p = 0.05	unpaired t-test
**P-tau positive cells**	Cis P-tau	3.8 ± 0.611	21.22 ± 1.37	p = 0.0003	unpaired t-test
	Trans P-tau	56.17 ± 2.8	48.07 ± 1.8	p = 0.074	unpaired t-test
	Gauche P-tau	70.83 ± 1.74	62.23 ± 2.92	p = 0.0674	unpaired t-test

Note: unpaired t-test Cis P-tau, Trans P-tau, and Gauche P-tau (Figure 2)

**Assay**	**Name of P-tau**	**mean ± SEM Control**	**mean ± SEM aging stress**	**P value**	**Test description**
**P-tau intensity**	Cis P-tau	0.66 ± 0.24	1.767 ± 0.28	p = 0.042	unpaired t-test
	Trans P-tau	1.074 ± 0.45	1.142 ± 0.04	p = 0.889	unpaired t-test

	Gauche P-tau	0.05 ± 0.01	0.4 ± 0.05	p = 0.004_	unpaired t-test
**P-tau positive cells**	Cis P-tau	29 ± 2	37.67 ± 1.45	p = 0.0248	unpaired t-test
	Trans P-tau	24.83 ± 2.8	23 ± 3.21	p = 0.693	unpaired t-test
	Gauche P-tau	20.27 ± 2.80	74.67 ± 8.66	p = 0.0039	unpaired t-test

Note: unpaired t-test Cis P-tau, Trans P-tau, and Gauche P-tau (Figure 3)

Table S2: Supplementary table with the titration data, which includes the various mAb concentrations tested and the corresponding dosages selected for the study.

**Gauche mAb Concentration (µM)**	**Cis mAb concentration (µM)**
0.0009	0.0009
0.001	0.001
0.0045	0.0045
0.009	0.009
0.01	0.01
0.03	0.03
0.06	0.06
