# Supplementary material for: A Novel Phosphorylated Tau Conformer Implicated in the Tauopathy Pathogenesis of Human Neurons
Source: Biomolecules. 2025 Apr 15;15(4):585. doi: 10.3390/biom15040585 (PMC12025006; doi:10.3390/biom15040585)

Sup. Fig. 4B

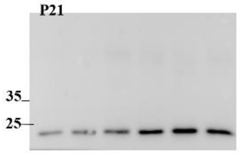

Sup. Fig. 3B

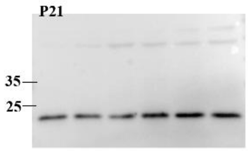

Figure 2C

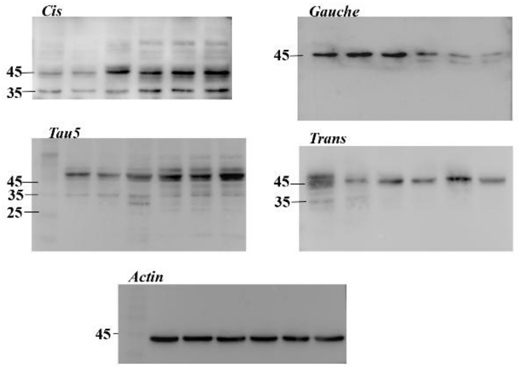

Electrophoresis gel

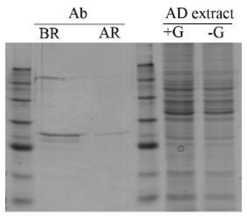

BR= Before resin
AR= After resin
G= Gauche
Ab= Antibody
AD= Alzheimer's disease

Figure 1C

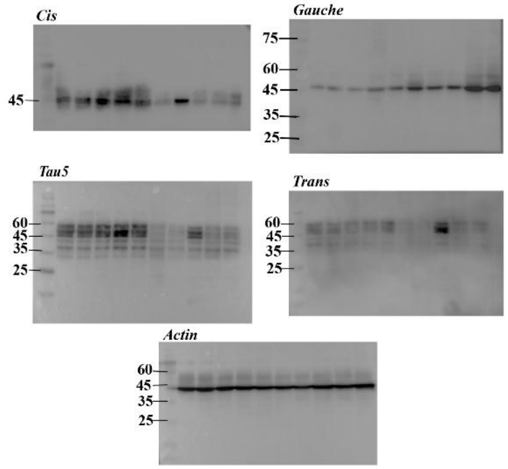

Figure 3C

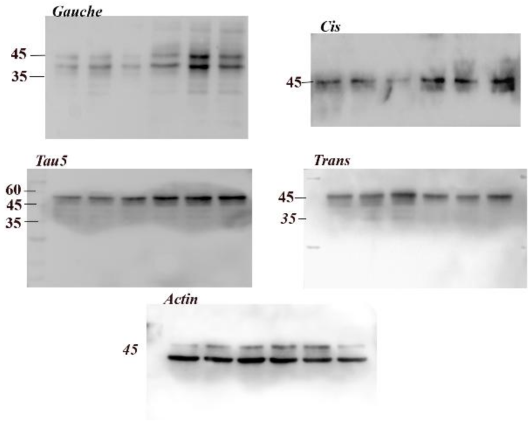

Supplement: Supplementary file 1 [file biomolecules-15-00585-s001.zip › biomolecules-3509752-WB.pdf]
